# Interleukin-13 Propagates Prothrombin Kringle-2-Induced Neurotoxicity in Hippocampi In Vivo via Oxidative Stress

**DOI:** 10.3390/ijms22073486

**Published:** 2021-03-27

**Authors:** Jae Yeong Jeong, Rayul Wi, Young Cheul Chung, Byung Kwan Jin

**Affiliations:** 1 Department of Biochemistry and Molecular Biology, School of Medicine, Kyung Hee University, Seoul 02447, Korea; jyoung0229@khu.ac.kr; 2 Department of Neuroscience, Graduate School, Kyung Hee University, Seoul 02447, Korea; rayul0912@khu.ac.kr; 3 Department of Predictive Toxicology, Korea Institute of Toxicology, Daejeon 34114, Korea

**Keywords:** interleukin-13 (IL-13), prothrombin kringle-2 (pKr-2), microglia/macrophages and neutrophils, oxidative/nitrosative stress, reactive oxygen species (ROS)

## Abstract

The present study investigated expression of endogenous interleukin-13 (IL-13) and its possible function in the hippocampus of prothrombin kringle-2 (pKr-2)-lesioned rats. Here we report that intrahippocampal injection of pKr-2 revealed a significant loss of NeuN-immunopositive (NeuN^+^) and Nissl^+^ cells in the hippocampus at 7 days after pKr-2. In parallel, pKr-2 increased IL-13 levels, which reached a peak at 3 days post pKr-2 and sustained up to 7 days post pKr-2. IL-13 immunoreactivity was seen exclusively in activated microglia/macrophages and neutrophils, but not in neurons or astrocytes. In experiments designed to explore the involvement of IL-13 in neurodegeneration, IL-13 neutralizing antibody (IL-13Nab) significantly increased survival of NeuN^+^ and Nissl^+^ cells. Accompanying neuroprotection, immunohistochemical analysis indicated that IL-13Nab inhibited pKr-2-induced expression of inducible nitric oxide synthase and myeloperoxidase within activated microglia/macrophages and neutrophils, possibly resulting in attenuation of reactive oxygen species (ROS) generation and oxidative damage of DNA and protein. The current findings suggest that the endogenous IL-13 expressed in pKr-2 activated microglia/macrophages and neutrophils might be harmful to hippocampal neurons via oxidative stress.

## 1. Introduction

Reactive oxygen species/reactive nitrogen species (ROS/RNS), such as superoxide anion (O_2_^−^), nitric oxide (NO), peroxynitrite (ONOO^-^) and hydrogen peroxide (H_2_O_2_), are byproducts during metabolic processes from endogenous sources including mitochondria, peroxisomes, and the endoplasmic reticulum [1,2,3]. Either excess production of ROS/RNS or a deficiency of antioxidants in the system cause oxidative/nitrosative stress, which can potentially cause damage to cellular biomolecules, including lipids and membranes, proteins, and DNA [2,4]. Especially in the central nervous system (CNS), regulating the oxidative/nitrosative stress is essential for brain cell survival and is associated with the initiation and progression of neurodegenerative disease, including Alzheimer’s disease (AD), Parkinson’s disease (PD), amyotrophic lateral sclerosis (ALS) and stroke [1,4,5,6]. Accordingly, regulation of molecular mechanisms of oxidative/nitrosative stress is still an unmet need for developing potential therapeutic targets against neurodegeneration.

Microglia, a subset of macrophages in the CNS, play a variety of roles in CNS-related immune response and the maintenance of brain homeostasis [7,8,9]. In pathological conditions, resting microglia develop into an activated state with amoeboid morphology and mediate the inflammatory responses associated with altered diverse cell-surface receptor expression [10,11], increased phagocytic activities [12], and production of proinflammatory cytokines and oxidative/nitrosative stress [13,14]. Furthermore, microglia are known to participate in ROS/RNS production during neurodegenerative process in AD and dementia [8,15].

Prothrombin kringle-2 (pKr-2) is the second domain of prothrombin and is derived from prothrombin to active thrombin. pkr-2 is known to induce microglial activation, ROS/RNS generation, and production of proinflammatory cytokines, such as tumor necrosis factor-alpha (TNF-α) and interleukin-1beta (IL-1β) in vivo and in vitro [16,17,18,19,20]. Levels of pKr-2 and thrombin were elevated in the postmortem brains of AD and PD patients [21,22], suggesting that pkr-2 might be involved in neuropathological processes as an endogenous neurotoxin. Regarding this, we also demonstrated that pKr-2-induced microglial activation and microglial proinflammatory cytokines cause neurodegeneration in rat cortices, substantiae nigrae, and hippocampi in vivo [16,17,18].

Interleukin-13 (IL-13) is a well-known anti-inflammatory cytokine produced by Th2 cells, mast cells, basophils, eosinophils, and B cells. IL-13 modulates the function of mast cells, macrophages, eosinophils, and B cells, and inhibits production of inflammatory cytokines [23,24]. In the CNS, microglia and neurons have been identified as the source of IL-13 [25]. IL-13 has a neuroprotective effect which works by modulating microglial polarity to an anti-inflammatory state [23,26] and promoting death of activated microglia [27,28]. By contrast, IL-13 can induce immune-mediated neurotoxicity through producing proinflammatory cytokines and ROS/RNS stress in activated microglia/macrophages and neutrophils, indicating that IL-13 is harmful to neurons [17,19,29]. Thus, the current study investigated whether activated microglia/macrophages and neutrophils-derived from IL-13 could contribute to pKr-2-induced hippocampal neuronal death by regulating oxidative/nitrosative stress in vivo.

## 2. Results

### 2.1. pKr-2 Induces Degeneration of Hippocampal Neurons and Activation of Microglia/Macrophages and Neutrophils in the Hippocampus In Vivo

**Figure 1:**
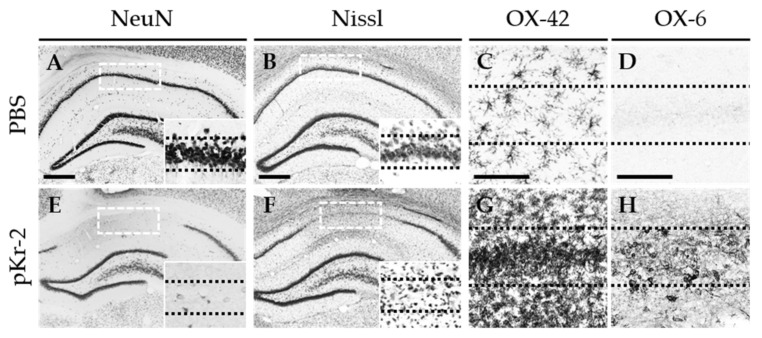
Prothrombin kringle-2(pKr-2) induces neurodegeneration and activation of microglia/macrophages and neutrophils in the CA1 area of the hippocampus in vivo. PBS (**A**–**D**) as a control or prothrombin kringle-2 (pKr-2; **E**–**H**; 48 μg/μL) was unilaterally injected into the CA1 layer of rat hippocampus. Animals were transcardially perfused and brains were prepared for immunohistochemical staining at 7 days after pKr-2 injection. Every sixth serial section was immunostained for NeuN (neuronal nuclei), Nissl, OX-42 (complement receptor 3, CR3) or OX-6 (major histocompatibility complex class II, MHC II). (**A**,**E**) NeuN immunostaining to identify neurons in the CA1 layer of hippocampus. Scale bar, 500 μm. (**B**,**F**) Nissl substance was stained in the CA1 layer of hippocampus. Scale bar, 500 μm. (**C**,**G**) OX-42 immunostaining to identify microglia/macrophages and neutrophils in the CA1 layer of hippocampus. Scale bar, 100 μm. (**D**,**H**) OX-6 immunostaining to identify activated microglia. Scale bar, 100 μm. Note the morphological changes of microglia/macrophages and neutrophils from resting state (small and ramified) to activated state (large, short, and thick) in the pKr-2-injected CA1 layer of hippocampus, compared with the PBS-injected control. Insets are magnified from rectangles (**A**,**B**,**E**,**F**). Dotted lines indicate the CA1 layer of the hippocampus (**A**–**H**).

pKr-2 (48 μg) or PBS as a control was unilaterally injected into the CA1 layer of the hippocampus of rats. Seven days later, the brains were removed, and sections were processed for immunostaining for neuronal nuclei (NeuN) to detect general neurons or Nissl staining. There was a considerable loss of NeuN-immunopositive (NeuN^+^) (Figure 1E) and Nissl-stained cells (Figure 1F) at 7 days in the pKr-2-injected hippocampus compared with PBS-injected controls (Figure 1A,B). In parallel, pKr-2-activated microglia/macrophages and neutrophils, visualized by immunohistochemical staining using antibody against the complement receptor type 3 (OX-42) (Figure 1G) and the major histocompatibility complex class II antigens (OX-6) (Figure 1H), were also observed in the CA1 layer of the hippocampus, where degeneration of hippocampal neurons was found compared with PBS-injected controls (Figure 1C,D). pKr-2 triggered profound activation of microglia/macrophages and neutrophils in the hippocampus, with enhanced staining and activated morphology (large cell bodies with short, thick, or no processes) in OX-42^+^ cells (Figure 1G) compared with PBS-injected control (Figure 1C) showing the resting state of OX-42^+^ cells (small cell bodies with ramified processes). These data also showed that pKr-2 treatment resulted in many OX-6^+^ cells in the CA1 layer of the hippocampus (Figure 1H), whereas few of OX-6^+^ cells were seen in PBS-treated controls (Figure 1D).

### 2.2. pKr-2-Induced IL-13 Expression Is Localized within Activated Microglia/Macrophages and Neutrophils in the Hippocampus In Vivo

**Figure 2:**
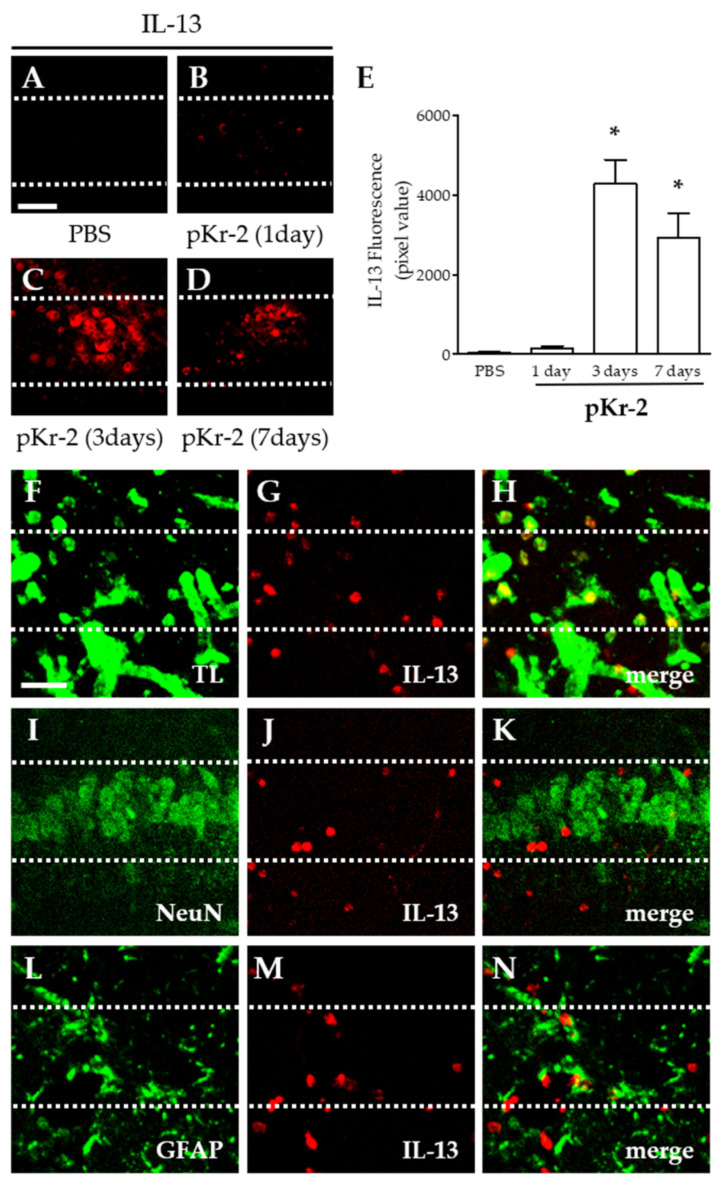
Endogenous expression of IL-13 in the CA1 layer of pKr-2-injected hippocampus in vivo. PBS (**A**) or pKr-2 (**B**–**D**,**F**–**N**) was unilaterally injected into the CA1 layer of rat hippocampi. At indicated time points, animals were transcardially perfused and brains were prepared for immunohistochemical staining. (**A**–**E**) Immunofluorescence images of endogenous interleukin-13 (IL-13) (**A**–**D**) and quantification (**E**) in the CA1 layer of rat hippocampus at indicated time points. Scale bar, 40 μm. * *p* < 0.001, significantly different from PBS (control). Mean ± SEM; *n* = 4 to 6 in each group, ANOVA and Newman–Keuls analysis. (**F**–**N**) Double immunofluorescence images of tomato lectin (TL; **F**, green) for microglia/macrophages and neutrophils and IL-13 (**G**, red) or NeuN (**I**, green) for neurons and IL-13 (**J**, red) or glial fibrillary acidic protein (GFAP; **L**, green) for astrocytes and IL-13 (**M**, red) and both images are merged (yellow, **H**,**K**,**N**) in the CA1 layer of hippocampus at 12 h after pKr-2 injection. Scale bar, 25 μm. These data are representative of four to five animals per group. Dotted lines indicate the CA1 layer of the hippocampus.

We examined whether intrahippocampal injection of pKr-2 induced expression of interleukin 13 (IL-13) in the hippocampus. Analysis by immunohistochemical staining demonstrated that pKr-2 induced IL-13 expression as early as 1 day after injection, with maximal levels reached 3 days after injection and sustained up to 7 days after injection (Figure 2B–E). In contrast to pKr-2-injected hippocampus, IL-13 expression was inconsiderable in PBS-injected hippocampus as control (1 day; Figure 2A,E).

To identify the cell type expressing endogenous IL-13, double immunofluorescence staining was performed in combination with IL-13 with tomato lectin (TL)^+^ microglia/macrophages and neutrophils, NeuN^+^ neurons, or glial fibrillary acidic protein (GFAP)^+^ astrocytes at 12 h after pKr-2 injection. Endogenous IL-13 was mainly expressed in TL^+^ microglia/macrophages and neutrophils (Figure 2F–H), but neither in NeuN^+^ neurons (Figure 2I–K) nor GFAP^+^ astrocytes (Figure 2L–N) in the CA1 layer of hippocampus in vivo.

### 2.3. Neurotoxic Action of IL-13 on Degeneration of Hippocampal Neurons via iNOS and MPO in the CA1 Layer of Hippocampus In Vivo

**Figure 3:**
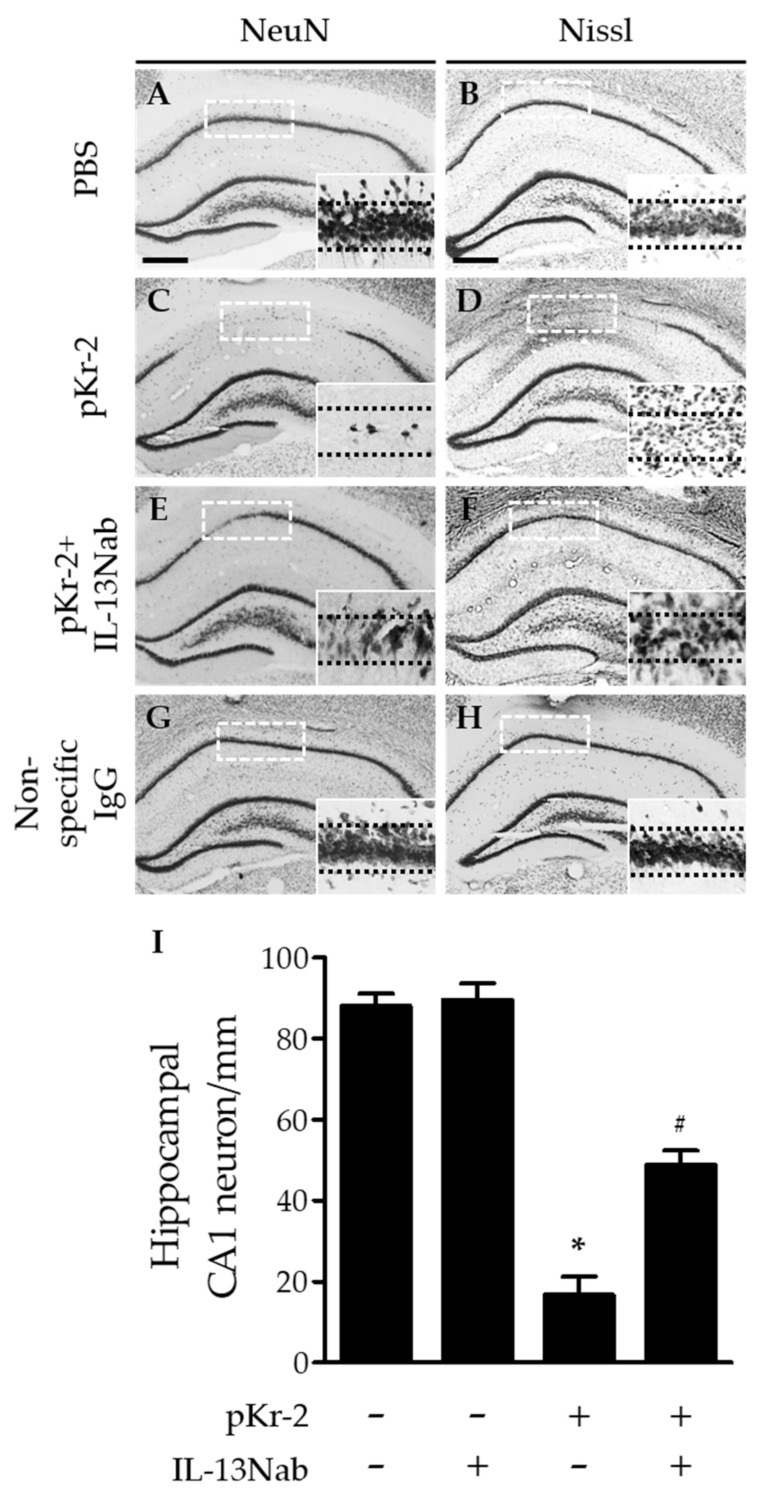
IL-13 contributes to neurodegeneration in the CA1 layer of hippocampus in vivo. PBS (**A**,**B**) or pKr-2 was unilaterally injected into the CA1 layer of hippocampus in the absence (**C**,**D**) or presence of IL-13 neutralizing antibody (IL-13Nab; **E**,**F**; 1 μg). Non-specific IgG (1 μg; **G**,**H**) was used as a control of IL-13Nab. Animals were transcardially perfused and brains were prepared for NeuN immunostaining and Nissl staining at 7 days after pKr-2 injection. (**A**,**C**,**E**,**G**) NeuN immunostaining to identify neurons in the CA1 layer of hippocampus. Scale bar, 500 μm. (**B**,**D**,**F**,**H**) Nissl substance was stained in the CA1 layer of hippocampus. Scale bar, 500 μm. (**I**) Quantification of NeuN-immunopositive (NeuN^+^) cells in the CA1 layer of pKr-2-injected hippocampus. * *p* < 0.001, significantly different from PBS (control). ^#^ *p* < 0.001, significantly different from pKr-2. Mean ± SEM; *n* = 4 to 6 in each group, ANOVA and Newman–Keuls analysis. Insets are magnified from rectangles and dotted lines indicate the CA1 layer of the hippocampus (**A**–**H**).

To elucidate the physiological functions of IL-13 mainly expressed in microglia/macrophages and neutrophils after pKr-2 injection, we examined whether pKr-2-induced degeneration of hippocampal neurons could be affected by treatment of IL-13 neutralizing antibody (IL-13Nab) for blocking the function of IL-13. For this purpose, IL-13Nab was unilaterally co-injected with pKr-2 into the CA1 layer of hippocampus (Figure 3E,F). Seven days later, NeuN immunostaining (A,C,E) and Nissl (B,D,F) staining demonstrated protective effects of IL-13Nab on hippocampal neurons in vivo. When NeuN^+^ cells on the ipsilateral side were quantified, it was found that IL-13Nab significantly increased the number of NeuN^+^ cells in the CA1 layer of the hippocampus compared with pKr-2 only (Figure 3I). As controls, IL-13Nab alone (Figure 3I) and nonspecific goat IgG in the absence (Figure 3G,H) or presence of pKr-2 [16] (data not shown) did not affect neuronal survival [16,19,29,30], similar to that observed with PBS or pKr-2 only.

**Figure 4:**
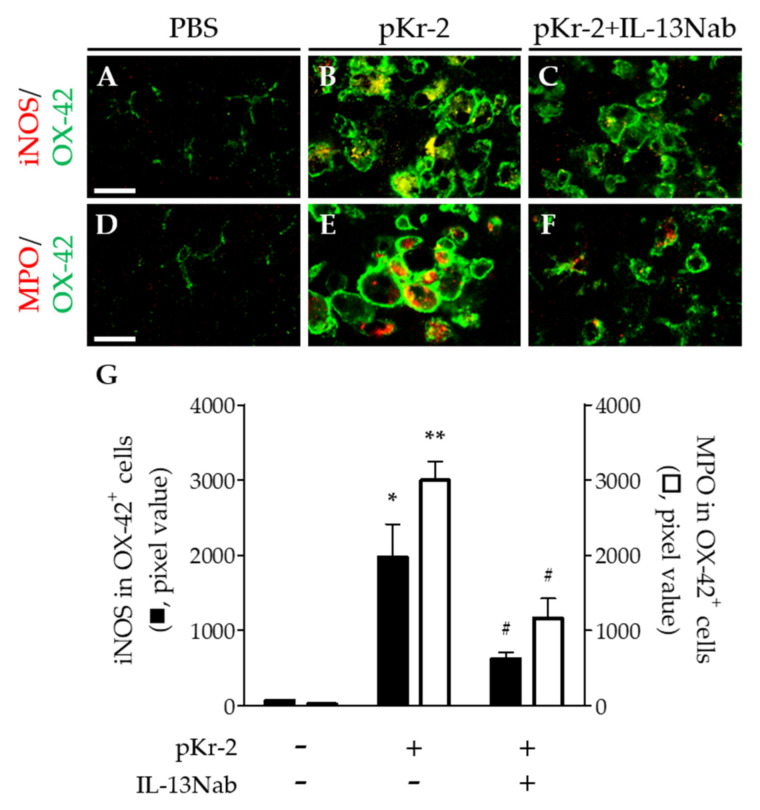
IL-13 is involved in expression of microglia/macrophages and neutrophils -derived myeloperoxidase (MPO) and inducible nitric oxide synthase (iNOS) in the CA1 layer of pKr-2-injected hippocampus in vivo. PBS (**A**,**D**) or pKr-2 (**B**,**C**,**E**,**F**) was unilaterally injected into the CA1 layer of hippocampus in the absence (**B**,**E**) or presence of IL-13Nab (**C**,**F**; 1 μg). Animals were transcardially perfused and brains were prepared for immunohistochemical staining at 3 days after pKr-2 injection. (**A**–**C**) Double immunofluorescence images of iNOS (red) and OX-42 (green) for microglia/macrophages and neutrophils and both images are merged (yellow) in the CA1 layer of hippocampus. Scale bar, 20 μm. (**D**–**F**) Double immunofluorescence images of MPO (red) and OX-42 (green) for microglia/macrophages and neutrophils and both images are merged (yellow) in the CA1 layer of hippocampus. Scale bar, 20 μm. (**G**) Quantification of iNOS (left y axis; black rectangle) or MPO (right y axis; white rectangle) expression in OX-42^+^ microglia/macrophages and neutrophils. * *p* < 0.01, ** *p* < 0.001, significantly different from PBS (control). ^#^ *p* < 0.01, significantly different from pKr-2. Mean ± SEM; *n* = 4 in each group, ANOVA and Newman–Keuls analysis.

Activated microglia/macrophages and neutrophils produce inducible nitric oxide synthase (iNOS) and myeloperoxidase (MPO), which induces oxidative/nitrosative stress, resulting in neurodegeneration [4,16]. Thus, we examined whether intrahippocampal injection of pKr-2 induced expression of iNOS and MPO in activated microglia/macrophages and neutrophils. pKr-2 (48 μg) or PBS as a control was unilaterally injected into the CA1 layer of the hippocampus of rats. Three days later, immunohistochemical analysis showed that pKr-2 significantly increased expression of iNOS (Figure 4B,G) and MPO (Figure 4E,G) within OX-42^+^ microglia/macrophages and neutrophils, respectively, compared to PBS-injected control (Figure 4A,D,G). To verify involvement of iNOS and MPO with IL-13-induced neurotoxicity, IL-13Nab was unilaterally co-injected with pKr-2 into the CA1 layer of hippocampus. Three days later, immunohistochemical analysis demonstrated that treatment of IL-13Nab significantly reduced pKr-2-triggered expression of iNOS (Figure 4C,G) and MPO (Figure 4F,G).

### 2.4. IL-13 Is Associated with Oxidative/Nitrosative Stress in the CA1 Layer of pKr-2-Injected Hippocampus In Vivo

**Figure 5:**
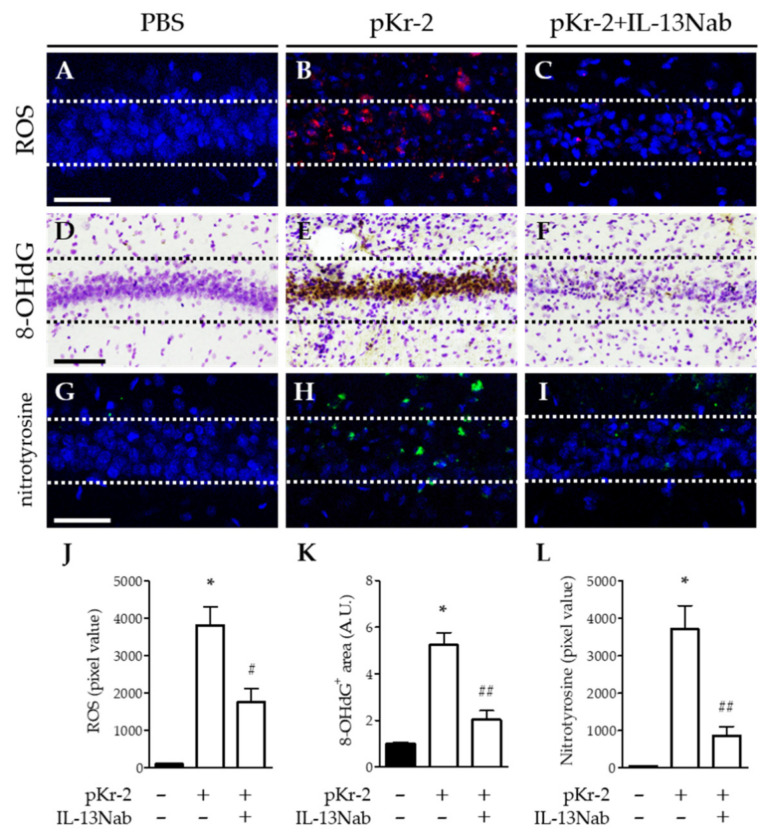
IL-13 modulates oxidative/nitrosative stress in the CA1 layer of pKr-2-injected hippocampus in vivo. Sections (**A**,**D**,**G**; PBS, **B**,**E**,**H**; pKr-2, **C**,**F**,**I**; pKr-2 + IL-13Nab) adjacent to those used in Figure 4 were processed for hydroethidine histochemistry or immunohistochemical staining. (**A**–**C**) Hydroethidine histochemistry to detect oxidant production (reactive oxygen species; ROS; red) in the CA1 layer of hippocampus. Nuclei were counterstained with DAPI (blue). Scale bar, 50 μm. (**D**–**F**) 8-OHdG immunostaining with Nissl staining to detect oxidative DNA damages in the CA1 layer of hippocampus. Scale bar, 100 μm. (**G**–**I**) Immunofluorescence images of nitrotyrosine immunostaining (green) to detect protein nitration in the CA1 layer of hippocampus. Nuclei were counterstained with DAPI (blue). Scale bar, 40 μm. (**J**) Quantification of ROS expression. * *p* < 0.001, significantly different from PBS (control). ^#^ *p* < 0.05, significantly different from pKr-2. Mean ± SEM; *n* = 4 to 5 in each group, ANOVA and Newman-Keuls analysis. (**K**) Quantification of 8-OHdG expression. * *p* < 0.001, significantly different from PBS (control). ^##^ *p* < 0.001, significantly different from pKr-2. Mean ± SEM; *n* = 4 to 5 in each group, ANOVA and Newman–Keuls analysis. (**L**) Quantification of nitrotyrosine expression. * *p* < 0.001, significantly different from PBS (control). ^##^ *p* < 0.001, significantly different from pKr-2. Mean ± SEM; *n* = 4 in each group, ANOVA and Newman–Keuls analysis. Dotted lines indicate the CA1 layer of the hippocampus (**A**–**I**).

We hypothesized that oxidative/nitrosative stress as a result of IL-13-stimulated expression of iNOS and MPO contributes to pKr-2-induced neurodegeneration in hippocampus in vivo. To test this, we investigated whether IL-13Nab altered the effects of pKr-2 on oxidative/nitrosative stress by analyzing levels of reactive oxygen species (ROS) (O_2_^−^ production), DNA oxidation, and protein nitration at 3 days post pKr-2. For O_2_^−^ production, oxidized hydroethidine (red fluorescent products) was analyzed by hydroethidine histochemistry. As a result, pKr-2 injection correlated with increased O_2_^−^ production in CA1 layer of hippocampus in vivo (Figure 5B,J), compared to PBS-injected control (Figure 5A,J). By contrast, treatment of IL-13Nab significantly reduced pKr-2-triggered O_2_^−^ production (Figure 5C,J). For DNA oxidation, we measured the levels of 8-OHdG at 3 days post pKr-2. Immunohistochemical analysis showed pKr-2-stimulated increase in levels of 8-OHdG (Figure 5E,K) compared to PBS-injected control (Figure 5D,K). Treatment with IL-13Nab significantly attenuated pKr-2-increased levels of 8-OHdG (Figure 5F,K). For protein nitration, analysis by nitrotyrosine immunohistochemistry revealed pKr-2-stimulated increase in protein nitration (Figure 5H,L) compared to PBS-injected control (Figure 5G,L). Treatment with IL-13Nab dramatically mitigated pKr-2-stimulated increase in levels of nitrotyrosine (Figure 5I,L).

## 3. Discussion

The cytokine IL-13 is secreted protein and can play both beneficial and detrimental roles in the CNS [23,26]. A study in humans with multiple sclerosis found that high levels of IL-13 in the cerebral spinal fluid might exert a neuroprotective effect by enhancing gamma aminobutyric acid over glutamate transmission [31]. Intracerebral injection of IL-13 reduced amyloid deposition and improved spatial learning and memory in an AD transgenic mouse model, indicating neuroprotection [32]. By marked contrast, IL-13 contributed to degeneration of cortical neurons in pKr-2-treated rat cortex [17] and nigral dopamine neurons in mice lacking IL-13 receptor alpha 1 [33], suggesting a neurotoxic action of IL-13. In addition, intrahippocampal injection of amyloid beta_1-42_ [19] and thrombin [29] produced IL-13-mediated neurodegeneration in CA1 layer of rat hippocampus. In the current study, blocking action of IL-13 by IL-13Nab results in survival of hippocampal neurons against pKr-2-induced neurotoxicity. These data suggest that endogenous IL-13 is an important determinant in pKr-2-induced neurotoxicity in the CA1 layer of hippocampus in vivo.

Microglia, resident immune cells of the CNS, are associated with a variety of neuropathologies [13,34,35]. Upon activation, microglia contribute to oxidative/nitrosative stress by producing considerable ROS/RNS, which cause the death of microglia as well as neurons [5,30,36,37,38]. Activated microglia can induce increased levels of iNOS, one of the major sources for excessive production of ROS/RNS and resultant oxidative damage to proteins, lipids, and DNA in neurodegenerative diseases [3,4,6]. Many in vivo studies, including ours, showed iNOS-induced generation of ROS/RNS in the cerebral cortex, substantia nigra, and hippocampus treated with thrombin, pKr-2, LPS, and beta-amyloid_1-42_ [17,19,39,40]. Accompanying the attenuation of neurotoxicity, treatment with IL-13Nab attenuated expression of iNOS, ROS/RNS, and oxidative damages in DNA and proteins in pKr-2-treated hippocampus in vivo, indicative of IL-13-induced oxidative/nitrosative damage in neurons. This is consistent with our findings that neutralization of IL-13 by IL-13Nab significantly reduced the expression of iNOS and/or ROS/RNS from activated microglia in pKr-2-treated cortex [17] and in hippocampus treated with thrombin- [29] or beta-amyloid_1-42_ [19] and the resultant survival of neurons in vivo.

MPO, a critical inflammatory enzyme, is present in infiltrated neutrophils, monocytes/macrophages, and neurons as well as activated microglia and astrocytes [5,41,42,43]. Activation of MPO can catalyze the reaction of chloride and H_2_O_2_ to produce hypochlorous acid (HOCl), which is a crucial cytotoxic factor and has oxidative activity able to react with DNA, proteins, and lipids [44,45]. MPO mediates oxidative stress by promoting the production of ROS/RNS, which can modulate the inflammation-related signaling pathways in microglia and cause oxidative damages to DNA, proteins and lipids, resulting in neurodegeneration in vivo [39,46,47,48,49,50,51]. Many reports, including ours, have shown that various antioxidants, such as compounds from medicinal herbs, capsaicin, and ethyl pyruvate, inhibited expression of MPO-derived ROS/RNS, reduced oxidative damage, and prevented neurodegeneration [42,52,53]. Our data demonstrated that treatment with IL-13Nab significantly mitigated pKr-2-induced MPO expression with attenuation of ROS/RNS levels and oxidative damages in the hippocampus in vivo, resulting in survival of neurons. This indicates that like the iNOS, IL-13 regulates the production of ROS/RNS derived from MPO.

In summary, neuroinflammation is associated with glial activation and glia-derived oxidative stress and inflammatory molecules, resulting in pathology of neurodegenerative diseases. Among them, oxidative/nitrosative stress (the imbalance between production and destruction of ROS/RNS) has been considered as a major risk factor of neurodegeneration. Here, we show that microglia/macrophages and neutrophils-derived from IL-13 plays a critical role on pKr-2-induced neurodegeneration of hippocampal CA1 areas in vivo. IL-13 neutralization attenuates levels of iNOS and MPO expression on microglia/macrophages and neutrophils, ROS production, and oxidative damages, resulting in neuronal survival. This suggests that endogenous IL-13 might be associated with neuropathological processes in which neuroinflammation and/or oxidative stress are involved.

## 4. Materials and Methods

### 4.1. Animals

Animal care, handling, and all experiments were performed according to the guideline set by Committee on Animal Research of Kyung Hee University (KHU-ASP-20-235; 30 June 2020). Female Sprague–Dawley rats were kept in temperature- (21–23 °C) and humidity-controlled conditions under a 12-h light/dark cycle. Throughout animal housing and the experiment, all animals had free access to food and water. All experiments were carried out to minimize animal suffering and the minimal number of animals necessary to produce significant scientific data were used.

### 4.2. Intrahippocampal Microinjection

For pKr-2 injection, rats (10-week-old. 240–260 g) were anesthetized by intraperitoneal injection of chloral hydrate (360 mg/kg; Sigma-Aldrich., St. Louis, MO, USA) and immobilized on stereotaxic instruments [16,17]. According to the atlas of Paxinos and Watson (ref), rats received unilateral injection of 48 μg pKr-2 (Haematologic Technologies., Essex Junction, VT, USA) in 4 μL of phosphate-buffered saline (PBS; Gibco., Palsey, UK) into the right CA1 of hippocampus (3.6 mm posterior to bregma, 2.0 mm lateral to the midline, 2.8 mm beneath the skull of the brain) with 30 gauge of Hamilton syringe at a rate of 0.2 μL per minute. For IL-13 neutralization [17,19], IL-13 neutralizing antibody (IL-13Nab; R&D system., Minneapolis, MN, USA) or 1 μg non-specific IgG (R&D system., Minneapolis, MN, USA) as a control together with pKr-2 were injected into the equivalent coordinate of right CA1 layer of hippocampus.

### 4.3. Immunohistochemistry (IHC) and Nissl Staining

Animals were transcardially perfused with physiological saline containing 0.5% sodium nitrate and heparin (10 U/mL) and fixed by 4% paraformaldehyde in 0.1 M phosphate buffer (pH 7.4). After post-fixing with 30% sucrose solution, rat brains were coronally cut at 40-μm-thick using sliding microtome. Serial coronal sections were processed for immunohistochemical staining as previously described [16,17]. Briefly, brain sections were rinsed with PBS 2 times, quenched in PBS containing 3% H_2_O_2_, rinsed with PBS 3 times, blocked in PBS containing 1% bovine serum albumin (BSA; Millipore Corporation., Kankakee, IL, USA) with 0.2% Triton X-100 (Sigma-Aldrich., St. Louis, MO, USA), and then incubated with primary antibodies at room temperature on a shaker overnight (Table 1). After rinsing in PBS containing 0.5% BSA 2 times, sections were incubated with biotin-conjugated mouse IgG (Seracare., Milford, MA, USA) for 1 h, rinsed with PBS containing 0.5% BSA 2 times, and processed with avidin-biotin complex kit (Vector Laboratories., Burlingame, CA, USA). After that, sections were visualized by the mixture of 0.05% 3,3′-diaminobenzidine tetrahydrochloride (DAB; Sigma-Aldrich., St. Louis, MO, USA) with 0.003% H_2_O_2_. Finally, labeled brain sections were mounted on adhesive microscope slides and dried. A series of sections were viewed under a bright-field microscope (Olympus Optical., Tokyo, Japan). The antibodies used for immunohistochemical staining (IHC) are shown below (Table 1).

For Nissl staining, brain sections were mounted on adhesive microscope slides and dried. After that, sections were stained with 0.5% cresyl violet acetate solution (Sigma-Aldrich., St. Louis, MO, USA), dehydrated and viewed under a bright-field microscope. 

### 4.4. Immunofluorescence (IF) Staining

Serial coronal sections (40-μm-thick) were processed for immunofluorescence staining as previously described [16,17]. Briefly, brain sections were mounted on adhesive microscope slides and dried. After that, sections were rinsed with PBS 3 times, blocked in PBS containing 1% BSA with 0.2% Triton X-100, and then incubated with primary antibodies at 4 °C overnight (Table 2). After rinsing in PBS containing 0.5% BSA 2 times, sections were incubated with the appropriate fluorescence-conjugated secondary antibodies for 1 h and rinsed with PBS 3 times. Finally, labeled brain sections were covered with the Vectashield mounting medium (Vector Laboratories., Burlingame, CA, USA) for fluorescence with DAPI and analyzed under a confocal microscope (Carl Zeiss., Oberkochen, Germany). The antibodies used for IF are shown below (Table 2).

### 4.5. Hydroethidine Histochemistry for Detecting O_2_^−^ and O_2_^−^ -Derived Oxidants

Hydroethidine (Dihydroethidium; DHE) histochemistry was performed to evaluate O_2_^−^ and O_2_^−^-derived oxidants production [16,17]. DHE freely permeate the cell membrane and react with superoxide anions to form a red fluorescent product (2-hydroxyethidium) [54]. At 3 days after pKr-2 injection, hydroethidine (1 mg/kg, reconstituted in PBS containing 1% dimethyl sulfoxide) (Invitrogen., Carlsbad, CA, USA) was intravenously injected into the rat tail vein. After 45 min, rats were transcardially perfused and fixed. After post-fixing, rat brains were coronally cut at 40-μm-thick using sliding microtome and mounted on adhesive microscope slides. The labeled brain sections were covered with the Vectashield mounting medium (Vector Laboratories., Burlingame, CA, USA) and the oxidized hydroethidine product (ethidium) was investigated by confocal microscope (Carl Zeiss., Oberkochen, Germany).

### 4.6. Image J Analysis

Imaging data obtained from bright-field microscope and confocal microscope were analyzed as pixel values using the Image J program (National Institutes of Health, Washington County, MD, USA). Image J was used to quantify the chromogenic signal intensity of images adjusted above the threshold to rule out unspecific background signal. For measurement of IL-13, ROS, 8-OHdG, and nitrotyrosine, images were transformed to 8-bit grayscale and adjusted at the endpoint of threshold histogram. Then the pixel value was quantified and normalized by unstained area. For measurement of iNOS and MPO within OX-42^+^ microglia/macrophages and neutrophils, the adjusted images of each channel are colocalized using a colocalization plugin, then the overlaid signal are quantified.

### 4.7. Statistical Anaylsis

Statistical analyses were performed using GraphPad Prism 5 (GraphPad Software., San Diego, CA, USA). Results are expressed as mean ± standard error of the mean (SEM). For statistical evaluation between two groups, *p* value was assessed by Student’s unpaired t-test. For comparison of multiple groups, *p* value was assessed by one-way of variance (ANOVA) with Newman–Keuls analysis. *p* < 0.05 was considered to indicate statistical significance.

## Figures and Tables

**Table 1:** Antibodies used for immunohistochemical staining (IHC).

Category	Antibody	Company	Cat. No.	Dilution Factor	Detection Target
Primary Antibody	NeuN	Merck	MAB377	1:1000	Neurons
	OX-42	Bio-rad	MCA275G	1:400	Microglia/ Macrophages/ Neutrophils
	OX-6	BD Bioscience	554926	1:400	Activated microglia
	8-OHdG	Jaica	MOG-100P	1:300	Oxidative DNA damage
Secondary Antibody	Biotin-conjugated anti-mouse IgG	Seracare	5260-0051	1:400	Mouse IgG

**Table 2:** Antibodies used for immunofluorescence staining (IF).

Category	Antibody	Company	Cat. No.	Dilution Factor	Detection Target
Primary Antibody	IL-13	R&D systems	AF1945	1:200	IL-13
	FITC-TL	Vector Laboratories	FL-1171	1:1000	Microglia/ Macrophage/ Neutrophils
	NeuN	Merck	MAB377	1:1000	Neurons
	GFAP	Sigma-Aldrich	G3893	1:500	Astrocytes
	OX-42	Bio-rad	MCA275G	1:400	Microglia/ Macrophage/ Neutrophils
	iNOS	BD Biosciences	610333	1:200	iNOS
	MPO	Dakocytomation	A0398	1:500	MPO
	Nitrotyrosine	Abcam	Ab7048	1:50	Oxidative protein damage
Secondary Antibody	FITC-conjugated anti-mouse IgG	Sigma-Aldrich	AP124F	1:500	Mouse IgG
	Fluorescein conjugated anti-mouse IgG	Vector Laboratories	FL-2000	1:300	Mouse IgG
	Cy3-conjugated anti-rabbit IgG	Sigma-Aldrich	AP132C	1:1000	Rabbit IgG
	Alexa Fluor 594-conjugated anti-goat IgG	Invitrogen	A11058	1:1000	Goat IgG

## Data Availability

The data used to support the funding of this study are all provided within the article.
